# A Cortical Attractor Network with Martinotti Cells Driven by Facilitating Synapses

**DOI:** 10.1371/journal.pone.0030752

**Published:** 2012-04-16

**Authors:** Pradeep Krishnamurthy, Gilad Silberberg, Anders Lansner

**Affiliations:** 1 Department of Numerical Analysis and Computer Science, Stockholm University, Stockholm, Sweden; 2 Nobel Institute of Neurophysiology, Department of Neuroscience, Karolinska Institute, Stockholm, Sweden; 3 School of Computer Science and Communication, Department of Computational Biology, Royal Institute of Technology (KTH), Stockholm, Sweden; The University of Plymouth, United Kingdom

## Abstract

The population of pyramidal cells significantly outnumbers the inhibitory interneurons in the neocortex, while at the same time the diversity of interneuron types is much more pronounced. One acknowledged key role of inhibition is to control the rate and patterning of pyramidal cell firing via negative feedback, but most likely the diversity of inhibitory pathways is matched by a corresponding diversity of functional roles. An important distinguishing feature of cortical interneurons is the variability of the short-term plasticity properties of synapses received from pyramidal cells. The Martinotti cell type has recently come under scrutiny due to the distinctly facilitating nature of the synapses they receive from pyramidal cells. This distinguishes these neurons from basket cells and other inhibitory interneurons typically targeted by depressing synapses. A key aspect of the work reported here has been to pinpoint the role of this variability. We first set out to reproduce quantitatively based on *in vitro* data the di-synaptic inhibitory microcircuit connecting two pyramidal cells via one or a few Martinotti cells. In a second step, we embedded this microcircuit in a previously developed attractor memory network model of neocortical layers 2/3. This model network demonstrated that basket cells with their characteristic depressing synapses are the first to discharge when the network enters an attractor state and that Martinotti cells respond with a delay, thereby shifting the excitation-inhibition balance and acting to terminate the attractor state. A parameter sensitivity analysis suggested that Martinotti cells might, in fact, play a dominant role in setting the attractor dwell time and thus cortical speed of processing, with cellular adaptation and synaptic depression having a less prominent role than previously thought.

## Introduction

The population of inhibitory interneurons comprises only 15–20% of neocortical neurons. Despite being a minority, interneurons are believed to play an important role in shaping network activity patterns by directly controlling the input/output of principal cells. Recent advances in single-cell recording techniques and molecular biology have led to an explosion of data and knowledge about these inhibitory cells - their morphology, synaptic connections, short-term plasticity and molecular characteristics [1]–[3]. Much less is known about the specific functional roles in network function played by the diverse inhibitory interneuron subtypes. Apart from the differences in morphology and synaptic targeting on pyramidal cells (PC), interneuron synapses also exhibit different synaptic short-term plasticity properties, from strongly depressing to strongly facilitating. Synaptic depression of glutamatergic synapses between pyramidal cells has a dominant effect in controlling firing rate [4]. Only very few cortical models of various phenomena like gamma oscillations, working memory, slow-wave sleep (SWS) oscillations etc take into account this dynamical nature of the synapses [5]. Most of them add details to the cell morphology keeping synapses, by contrast, static [6]–[10].

We have previously developed and characterized a network model of neocortical layers 2/3. This cortical network model has a modular hypercolumnar structure in which each hypercolumn comprises a set of minicolumns. Such a module operates like a soft winner-take-all network typically allowing just one active minicolumn at a time. It operates as an attractor type associative memory and displays bistable irregular low-frequency firing and various phenomena like, e.g. pattern retrieval, completion and rivalry as well as spontaneous wandering of network between stored states [11]–[13]. Mounting experimental evidence shows that ongoing activity in cortex can exhibit complex spatiotemporal patters [14]–[16]. These patterns seem to wander among a set of intrinsic cortical states that reflects the overall cortical architecture. Also, using voltage sensitive dye imaging Kenet et al. revealed that in primary visual cortex these cortical states matched the functional map of orientation columns [17]. We here demonstrate how adding one type of inhibitory interneuron, the Martinotti cell (MC), to our previous network model affects the attractor network dynamics during spontaneous reactivation.

A computational study undertaken by Melamed et al. (2008) [18] showed that slow oscillations emerge from the interplay of excitatory and inhibitory populations. Like MCs, their inhibitory population received facilitating synapses from the excitatory population and it was demonstrated that such inhibition underlies the switching between *up* and *down* states. This study was based on a rate-based model with non-adapting excitatory cells connected by static synapses and utilized only one type of interneuron. A recent experimental study on cortical slices has also suggested that interneurons receiving facilitating synapses in neocortex might play a key role in the termination of *up* states [19].

The objective of the present study is to study the impact of inclusion of a late-onset interneuron, that is MC, in our attractor network model by investigating how it affects activity levels and attractor dwell time during spontaneous activity. Although an earlier study has shown the effects of late-firing MC in a firing rate model, ours is the first study that uses spiking units with PC-MC characteristics matched to *in vitro* data. Here we use single-compartmental Hodgkin-Huxley (HH) type neuron models [20] of two different types of interneurons embedded in a population of pyramidal cells with the ratio of pyramidal to interneuron being 90∶10. We have included dynamic synapses throughout the network, enabling us to show how pyramidal cells differentially excite interneurons via depressing and facilitating synapses. We commenced with reproducing in our model the PC-MC sub-circuit, as previously described by Silberberg & Markram (2007) and Silberberg (2008) [21], [22], and reproduced (a) frequency dependent disynaptic inhibition of pyramid cells, and (b) frequency dependent recruitment of MCs. Thereafter, we integrated this microcircuit in our cortical attractor network model [11]–[13] to study the dynamic effects on a more global scale. We addressed the effect of MCs on the attractor dwell time when the network operated without external input, thus freely “hopping” between the stored states. We show that basket cells (BC) that receive depressing excitatory synapses have a high firing rate at the beginning of the attractor state which then tapers off. On the other hand, MCs that receive facilitating synapses display a late onset of activation and tend to terminate an ongoing attractor state. We have further shown how the dwell time and peak firing of PCs varies with PC- MC connection density. An earlier computational study [12], in agreement with others [9], [23], [24], [25], [26], [27] had demonstrated spike-frequency adaptation and synaptic depression contributing to termination of attractor states. We here show how the MC activation could have an even stronger contribution to the termination of the attractor states relative to spike-frequency adaptation and synaptic depression between PCs.

## Methods

### Model Neurons

The cells included are layer 2/3 pyramidal cells (PC) and two different types of inhibitory interneurons. They are soma targeting horizontally projecting basket cells (BC) [28] and dendrite targeting, vertically projecting Martinotti cells (MC) that establish a disynaptic inhibitory feedback pathway between the pyramidal cells [22], [29].

PCs are of regular firing type. In the previous simulations, adaptation was modeled by calcium entering via voltage gated Ca-channels and activation of K_Ca_ channels. Here adaptation is modeled using the M-current, a slow non-inactivating potassium current described by Yamada et al. [30]. BCs are modeled as non-adapting, relatively fast-spiking cells. MCs have the same properties as BCs except that they are somewhat more adapting [22]. The multi-compartmental cells used in the previous work have here been replaced by single-compartment cells with size of each cell type’s soma, steady-state current and voltage equations, and conductance values taken from Pospischill et al. 2008 [31] (Table 1).

**Table 1:** Neuron parameters.

Parameter	Pyramidal	Basket	Martinotti	Unit
E_leak_	−70	−70	−70	mV
E_Na_	50	50	50	mV
E_K_	−100	−100	−100	mV
g_leak_	0.0001	0.00015	0.00015	S/cm^2^
g_Na_	0.05	0.05	0.05	S/cm^2^
g_K_	0.005	0.01	0.01	S/cm^2^
g_M_	7e^−5^	0.000098	0.0001	S/cm^2^
Soma diameter	96	67	67	µm
c_m_	1	1	1	µF/cm^2^

Single-compartment Hodgkin-Huxley model parameters for different classes of cortical neurons taken from Pospischill et al. (2008).

All models described here were single-compartment neurons (cylinder of diameter d and length L) described by the following membrane equation:
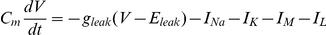
V  =  membrane potential, C_m_  =  specific capacitance of the membrane, g_leak_  =  specific resting (leak) membrane conductance, E_leak_  =  resting membrane reversal potential.

The kinetic parameters of the voltage-dependent *Na* current is given by
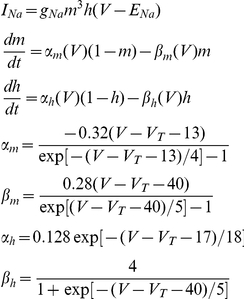
where g_Na_ and E_Na_ of different cortical cells are given in Table 1.

The kinetic parameters of the voltage-dependent *K* (delayed rectifier) current is given by
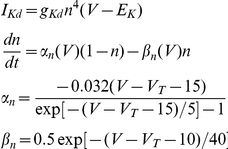
where g_Kd_ and E_Kd_ of different cortical cells are given in Table 1.

The kinetic parameters of the voltage-dependent *M* current is given by
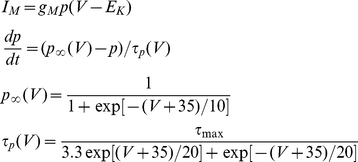
where g_M_ and τ_max_ of different cortical cells are given in Table 1.

We have used a point-conductance model of synaptic noise to account for the stochastic variation of conductance due to synaptic background activity on all cell models [32]. Table 2 gives parameters for mean conductance (g_e0_ and g_i0_) and standard deviation (σ_e_ and σ_i_). The level of this background noise is adjusted to a low firing rate on all cells (0.25 – 0.5 Hz).

**Table 2:** Synaptic noise parameters for each cell type from Destexhe et al. (2001).

	PC	BC	MC	Unit
g_e0_	0.0000121	0.000011	0.000011	µmho
g_i0_	0.00021	0.00002	0.00002	µmho
σ_e_	0.0018	0.0009	0.0009	µmho
σ_i_	0.0007	0.0003	0.0003	µmho

### Model Synapses

Glutamatergic synapses work on two broad categories of receptors: kainate/AMPA and NMDA. A mix of both provides the PC-PC glutamatergic transmission, but the PC-BC glutamatergic transmission is purely kainate/AMPA [33]. It is inconclusive from experiments whether PC-MC glutamatergic tranmission is plainly kainate/AMPA or a mix. For simulations presented in this paper, it is entirely kainate/AMPA. The GABA-ergic transmission in our model is exerted solely by GABA_A_ [33] (See Table 3). AMPA and GABA_A_ currents are given by [34], [35]:
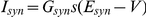
where the gating variable s (the fraction of open channels) is described by first-order kinetics via two equations:
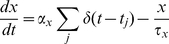
(1)

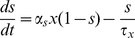
(2)


**Table 3:** Synapse parameters.

Pre-Post	Type	EPSP/IPSP amplitude (mV)	Rise time (s)	Delay time (s)	E_rev_ (mV)
PC-PC (local)	Kainate/AMPA	1.2	0.05	0.006	0
PC-PC(local)	NMDA	0.6	0.005	0.150	0
PC-PC(global)	Kainate/AMPA	0.2	0.05	0.006	0
PC-PC(global)	NMDA	0.2	0.005	0.150	0
PC-BC	Kainate/AMPA	1.8	0.05	0.006	0
PC-MC	Kainate/AMPA	0.2	0.05	0.006	0
BC-PC	GABAa	0.9	0.05	0.006	−75
MC-PC	GABAa	0.5	0.05	0.006	−75

The NMDA current is given by:
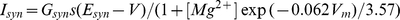
The gating variable *s* obeys the same types of equations (1,2). We have taken *τ_x_*  =  0.05 ms and *τ_s_*  =  6 ms for AMPA and GABA_A_, *τ_x_*  =  5 ms and *τ_s_*  =  150 ms for NMDA, α_x_  =  1 (dimensionless) and α_s_  =  1 (ms^−1^) for AMPA, NMDA and GABA_A_ [5], [35].

Short-term depression and facilitation were incorporated for all glutamatergic and GABAergic synapses [36], [37]. Every presynaptic spike, occurring at time t_sp_, causes a fraction *U* of the available pool to be utilized, the rate of return of resources given by τ_rec_, is multiplied by a quantity R (the fraction of available vesicles). R obeys the dynamical equation [38]:
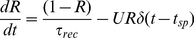
(3)The short-term depression is introduced into the synapse model by multiplying *α_x_* in (1), which mimics the transmitter release per spike, by R in (3) which is the fraction of available vesicles.

In modeling a facilitating synapse, *U* becomes a dynamic variable increasing at each presynaptic spike and decaying to the baseline level in the absence of spikes.
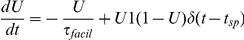
where *U*1 is a constant that determines the step increase in *U* and τ_facil_ is the decay time constant of facilitation.

At most three parameters completely define each connection type; *U*, τ_rec_ and τ_facil_ (depressing) or *U*1, τ_rec_ and τ_facil_ (facilitating). On one hand, there is no consensus on how precise these values should be. On the other hand, experiments do conform on a range of values [1]. The traces and parameters fitted to the model provided by Gilad Silberberg from his own experimental studies were very useful in setting these values. Particularly useful were the short-term dynamics between PC - MC and MC - PC connections. A connection with a high ‘U’ factor means the synapse has a strong ‘postsynaptic punch’ for initial spikes followed by a rapid depression. A low ‘U’ factor means lower initial release probability and thereby low initial impact on the postsynaptic side saving the transmitters for future presynaptic spikes. A synapse’s effect can be strongly depressing (τ_rec_>> τ_facil_) or strongly facilitating (τ_rec_ << τ_facil_) or intermediate displaying combined depressing-facilitating behaviour. The values assigned for each connection can be seen in Table 4.

**Table 4:** Short-term plasticity parameters inferred from Gupta et al. (2000) and traces provided by Silberberg G.

Pre-Post	Type	*U*	*U*1	τ_rec_	τ_facil_
PC-PC	Depressing	0.4	−	600	0
PC-BC	Depressing	0.5	−	600	0
PC-MC	Facilitating	−	0.05	20	1000
BC-PC	Depressing	0.25	−	500	50
MC-PC	Depressing	0.25	−	500	50

**Figure 1:**
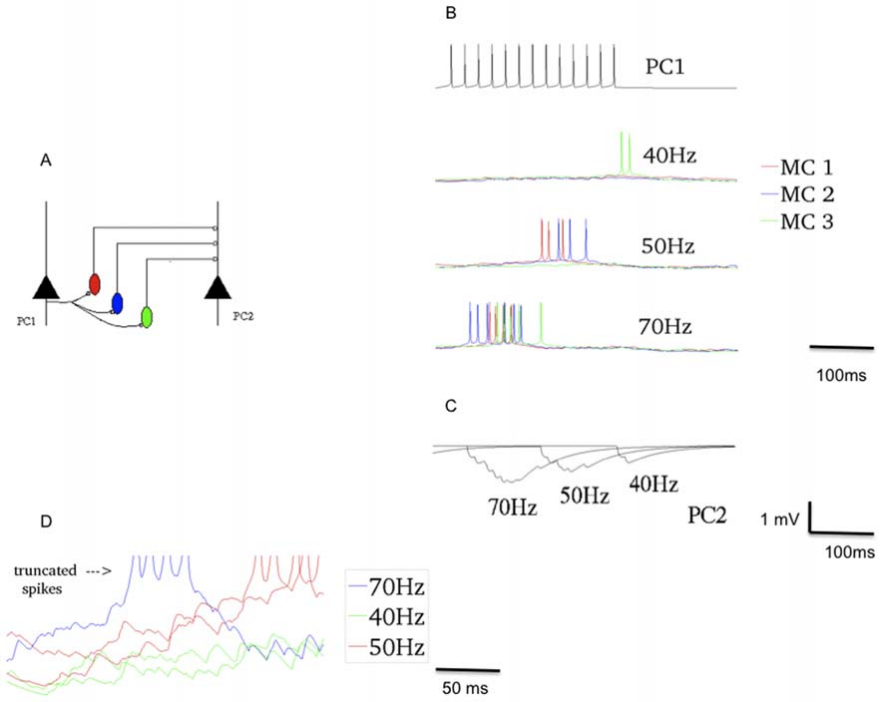
Frequency-dependent discharge of Martinotti cells (MCs). (A) Microcircuitry modeled as described in Silberberg & Markram (2007) showing the disynaptic pathway between pyramidal cells (PCs) (black) mediated by 3 MCs (red, blue and green). The PC1 to MC excitatory synapses are facilitating. (B) The presynaptic PC1 was stimulated by a train of APs at different frequencies (40, 50 and 70 Hz), shown for a 40 Hz input. The overlaid voltage traces of 3 post-synaptic MCs are shown. Firstly, higher frequency evoked post-synaptic APs with higher probability and shorter onset latency. Secondly, higher frequency recruited more intermediate MCs, in accordance with experiments (Silberberg & Markram, 2007). Rarely do all 3 MCs discharge for 40 Hz input, 2 MCs discharge in ninety percent of the trials during 50 Hz and all 3 MCs discharge in ninety percent of the trials during 70 Hz. The MC membrane potential jitter is due to the presence of background activity. (C) The increase in amplitude and decrease in latency of disynaptic response on PC2 membrane potential as a function of presynaptic AP train frequency. The monosynaptic excitation between pyramidal cells is turned off to present how the disynaptic response of MCs in experiment and model coincide. (D) Individual traces of MCs receiving synaptic input from PCs demonstrating membrane depolarization following different presynaptic discharge frequencies.

We used Thomson et al. (2002), Silberberg and Markram (2007) and Douglas and Martin (2004) data for assigning PC - PC (local and global), PC - interneuron and interneuron - PC connection strengths and their respective postsynaptic potential (PSP) amplitudes (See Fig.1) [22], [28], [39].

### Simulation

The computation model was run using the NEURON simulator [40]. Simulations were typically performed on 128 nodes of the Blue Gene/L computer at the Center for Parallel Computers at KTH. It took 70 seconds to simulate one second of network activity.

### Architecture of the Network Model

**Figure 2:**
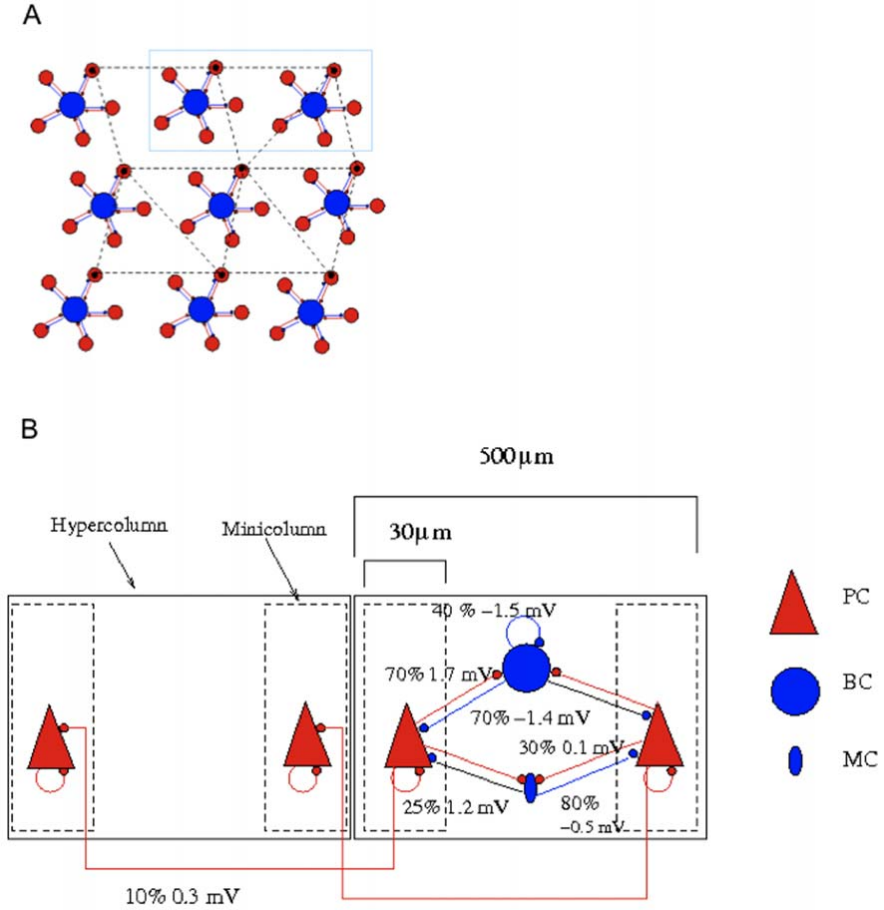
Schematic of the network arrangement and all the excitatory and inhibitory pathways between different cell types and their connection densities in the model. (A) Cartoon of a network of 9 hypercolumns with 5 minicolumns each. The model used had 36 hypercolumns. Each hypercolumn has 5 circularly arranged minicolumns. A minicolumn, represented by red discs, contains 30 densely connected (25%) PCs denoting local re-entry. The minicolumns in a hypercolumn receive inhibition from the cell population represented by blue discs, the excitatory (red) and inhibitory synapses (blue) are also shown. Dashed lines show minicolumns that are connected and distributed in different hypercolumns, which forms a pattern. (B) A small segment of the network blown-up to show the particulars, only here we see each blue disc houses 2 inhibitory cell types.

A detailed description of our full-scale conceptual model can be found in Djurfeldt et al. (2006) and Lundqvist et al. (2006) and the latest developments are found in Lundqvist et al. (2010) [12], [13], [41]. The sub-sampled neocortical model used here represents a 3×3 mm patch of cortex arranged on a square topology of 6×6 hypercolumns each separated by 500 µm, in agreement with hypercolumn diameter data from cat, i.e., 300–600 µm [42]. Each hypercolumn further constitutes several minicolumns – various estimates suggest that there are about one hundred minicolumns bundled into a hypercolumn [43]. In the current sub-sampled network model we have 5 minicolumns. The arrangement of cells in the local microcircuit together with connection probabilities and strengths (PSP amplitudes) are shown in Fig. 2. Each minicolumn (red disc) consists of 30 PCs densely connected to other PCs in the same minicolumn (25%) [44], [45], [46] and two regular spiking non-pyramidal (RSNP) interneurons (possibly double-bouquet cells) (not shown). Each hypercolumn has 8 BCs (circular blue disc). Each PC in a minicolumn targets 70% of its neighboring BCs and each BC targets 70% of the neighboring PCs. In the recent versions of the model, we have also introduced synaptic connections between BCs (40%) in the same hypercolumn. Even though electrical coupling between BCs have been observed, we have not included those in our model [47], [48]. The long-range minicolumn - minicolumn inhibition through RSNP cells, used in the previous study [12], is turned off here, since the dwell time of the attractor, which we measure here is not affected by its presence. Our new addition to this model is the MC pool (3 per hypercolumn, oval blue disc). The PC -> MC and MC -> PC connections show high convergence and divergence [49]. In our model, each MC receives input from 40% of the PCs in the hypercolumn and contacts 80% of the PCs in the hypercolumn [22], [49]. The extent of BC and MC inhibition is limited to the home hypercolumn. The minicolumns in a hypercolumn altogether sweep a width of approximately 100 mm and hence we have made two assumptions. (*a)* The extent of inhibition of horizontally projecting BCs and vertically projecting MCs may vary in the real cortex, but in this subsampled network, all the minicolumns lie within the reach of the BC and MC pool. (*b)* The PCs in each minicolumn target 8 neighboring BCs and 3 neighboring MCs. Since each hypercolumn has closely spaced adjacent minicolumns, all minicolumns share the same BC and MC pool. If we had not clustered minicolumns like it is done here, we could not have assumed just one pool.

The cartoon in Fig. 2 shows how the minicolumns in different hypercolumns, denoted by dashed lines, are connected. Thus, a set of minicolumns distributed over different hypercolumns represents a stored pattern or memory or an attractor of the network dynamics. In each hypercolumn, via lateral inhibition of BCs, the activity in an attractor state engages only one minicolumn (orthogonal patterns). In this network, consequently, we store as many patterns as the number of minicolumns in a hypercolumn. But by allowing overlapping memory patterns the number of patterns stored can be increased significantly [50].

## Results

### PC - MC Microcircuitry

We set out to reproduce how discharge of an individual PC at different rates induces differential delays in the discharge of MCs and how this influences a second PC. Fig. 1a shows the connection setup of this disynaptic inhibitory pathway involving two neighboring PCs and three intermediate MCs. High frequency activation of PCs is shown to exert inhibition in a significantly larger number of PCs by a supralinear increase in the recruitment of MCs [29]. To this end, we included synaptic background activity to show frequency dependent recruitment of MCs.

Connections from PCs to MCs are facilitating (*U*1  =  0.05, τ_rec_  =  20 ms, τ_facil_  =  1000 ms) [18]. Presenting an AP train in a presynaptic PC1 thus evokes a discharge in a post-synaptic MC with a delay and a delayed inhibition of PC2, which is postsynaptic to the MC. The latency of this discharge onset depends upon presynaptic stimulation frequency, as shown in slices [22]. Since the PC - MC connections are facilitating, they are endowed with low initial release probability, long facilitation time constants, short depression time constants (see Table 4), and small unitary EPSPs (0.3 mV on average). The voltage traces of all 3 MCs to 40, 50 and 70 Hz are overlaid in Fig 1b. When PC firing is 40 Hz, only one MC discharged in ninety percent of the trials; 2 MCs responded during 50 Hz in most of the trials and all 3 discharged readily during 70 Hz in every trial. Thus a jitter introduced in the membrane voltage overtly demonstrated a presynaptic frequency based recruitment of MCs (see Fig. 1d).

Connections from MCs to PCs displayed synaptic depression (*U* = 0.3, τ_rec_  =  500 ms, τ_facil_  =  5 ms) [1]. Monosynaptic IPSPs of these connections, as reported, have a small amplitude (–0.6 mV). Disynaptic responses on PC2 for different frequencies (40, 50 and 70 Hz) are shown in Fig. 1c. It demonstrates how the disynaptic responses of the model neuron increased in amplitude and decreased in latency as a function of presynaptic AP train frequency in accordance with Silberberg & Markram (2007) [22]. The change in amplitude of the disynaptic response from a 40 Hz presynaptic AP train to a 70 Hz AP train was reported nearly twice as high and intermediate for a 50 Hz AP train. In our model, we were able to fit this relationship quantitatively, in the presence of a jitter, when we assumed three MCs.

### MCs in a Network

We were then interested to investigate the effect of including MCs in our earlier attractor network model [12]. Previous studies showed how this network is capable of performing basic attractor network operations like pattern completion and pattern rivalry. Our focus now is to investigate the dynamic effects of MCs on the network activity, in the absence of any sensory-like external input.

**Figure 3:**
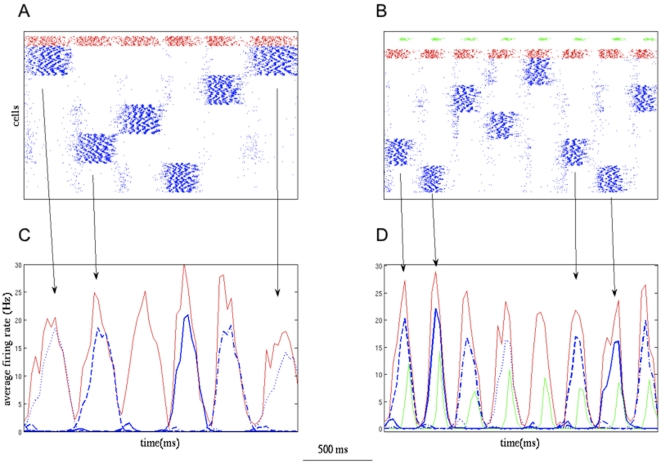
The activity of cells in the network. The output of different cell types are colour-coded for the sake of clarity; PCs (blue), BCs (red) and MCs (green). (A,C) Rastergram and average discharge rate of PCs and BCs versus time when inhibition from MCs is turned off. (A) When the network is subjected to a low background noise (0.25 – 0.5 Hz), it started hopping through the stored attractor states. PCs that form a pattern and are active (near) synchronously in different hypercolumns are grouped for visual aid. The x axis represents time, while the y axis represents neuron label. A dot in the rastergram means a spike of a neuron y at time x. (C) The time varying firing rate of all the cells is not evident in the rastergram. Average discharge rate versus time, bin size of 25 ms, makes it clearer. BCs fire at every attractor cycle since they receive excitation from all minicolumns in a hypercolumn. PCs from different patterns, represented by various blue line-strokes, took turns getting active. BCs, keeping step with PCs, had a high firing rate at the beginning of the attractor and tapered off maintaining the excitation - inhibition balance. (B, D) Same as above after the inclusion of MCs inhibition. (D) The late activity of MCs is apparent in the average firing rate. BCs with their characteristic depressing synapses are the first to respond (red). MCs receiving facilitating synapses discharge with a delay (green), and similar to BCs, as mentioned above, are active at every attractor cycle. MCs due to their strong projection to the neighbouring PCs within 100 µm radius (Silberberg & Markram, 2007), shut the activity thereby shifting the excitation - inhibition balance. Thus, presence of MCs inhibition controls the dwell time of the attractor.

Initially, we performed simulations omitting MCs. When the network was subjected to a low level of background noise, it started visiting various stored states randomly (Fig. 3a, top). The raster plot clearly shows how the PCs in different hypercolumns that form a pattern were (nearly) synchronously active. The rate of switching is about three states per second. The neurons that are active when the network engages in an attractor state receive stronger synaptic input, raised average membrane potential and an increase in spike rate, in agreement with the previous studies [12]. These concurrently active cells through local recurrent connections and global long-range connections maintain the persistent activity. Mean firing rate is a good measure to ascertain if the network has indeed entered an attractor state, and inquire into time evolution of PC and BC firing rates (Fig. 3a, bottom). It is calculated by setting a time window of 25 ms and counting the number of spikes that occurred in this time window, dividing this by the length of the time window and the number of active PCs. PCs fire (blue curve) briskly at the beginning of the attractor visit and the firing rate decreases owing to spike-frequency adaptation and synaptic depression (see below). Termination of an active attractor gives way to the activation of silent attractors, this cycle repeats *ad infinitum*. BCs fire at every attractor cycle since they are connected to all the minicolumns. Also, the BCs adjust their firing (red curve) in synchrony with PCs showing how excitation and inhibition during cortical activity is always in a state of dynamic balance [51].

MCs were introduced using the following connection paradigm: Each MC receiving input from 40% of PCs in a minicolumn and projecting back to 80% of PCs in a minicolumn. Below we discuss different connection paradigms. With the inclusion of MCs, the attractor state experiences early termination (Fig. 3b, top) resulting in an increased frequency in state transitions (from 2 Hz to 5 Hz). This early termination is due to delayed inhibition provided by MCs. The raster plot is similar to above, with spikes from MCs (green dots) included. The late firing of MCs is not conspicuous in the raster plot, so we instead calculated average discharge rates (Fig. 3b, bottom). The BCs that receive depressing synapses and high unitary EPSPs are the first to respond and provide inhibition throughout the attractor state (red curve) just like above. The MCs that receive low unitary EPSPs, characteristic of facilitating synapses, starts to engage with a delay after the onset of attractor activity (green curve), and thereby sweeping away the activity [52]. This is due to high divergence of MCs on its neighboring PCs, also shown in slices [22], [49]. Similar to BCs, MCs also fire at every attractor cycle since they are shared by all minicolumns in a hypercolumn.

### Different PC to MC Connection Strategies

MCs can be integrated into our network model in different ways compatible with experimental data. Here we investigated three different connection paradigms between PCs and MCs. We kept the MC → PC connections, which shows high divergence (80%), constant varying only the PC → MC connections. The different connection paradigms are (Con/Div): (15/80, 40/80, 80/80), where Con stands for convergence, and Div stands for divergence. For instance, if we assume the 40/80 paradigm, each MC receives converging input from 40% of presynaptic PCs in a minicolumn and diverges onto 80% of postsynaptic PCs in a minicolumn. If a PC exerts inhibition on another PC via 1 MC, 2 MCs and 3 MCs, we call it Type 1, Type 2 and Type 3 respectively. Going back to the microcircuit in Fig. 1, we find Type 1, Type 2 and Type 3 when the input frequency is 40, 50 and 70 Hz respectively. Table 5 contains the average number of Type 1, Type 2 and Type 3 connections in each connection paradigm.

**Table 5:** Different connectivity paradigms between PCs and MCs.

Con/Div	Type 1	Type 2	Type 3
15/80	97%	2%	1%
40/80	7%	87%	6%
70/80	2%	18%	80%

Type 1 if a PC exerts inhibition on another PC via one MC, Type 2 and Type 3 if it is via two and three MC respectively.

**Figure 4:**
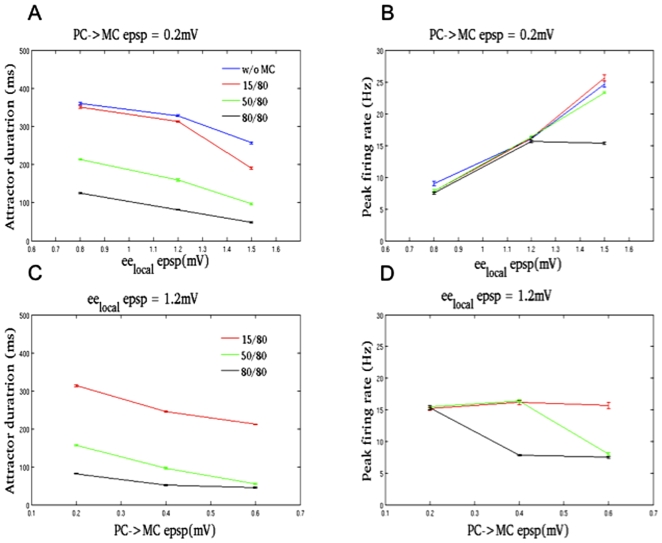
Attractor duration and peak firing rate of PCs during different connection paradigms when connection strengths are varied. See the text for a description of these connection paradigms. The value at the bottom of each subfigure is the varying quantity and on the top is the quantity that is held constant. (A,C) PCs attractor duration showed a linear response to increase in PC -> PC (0.8, 1.2, 1.5 mV) and PC -> MC (0.2, 0.4, 0.6 mV) epsp size. (A) The attractor duration decreased as PC -> PC strength increased. The response to “without MC” paradigm was similar to “15/80”, but “50/80” and “80/80” paradigms showed marked reduction in the attractor duration. (C) Increase in PC -> MC connection strength also showed a similar trend. (B,D) It takes about 80 ms after PCs enter an attractor state before spike-frequency adaptation and synaptic depression take effect causing a reduction in average firing rate. (B) If the MCs become active before the above factors take effect, the peak-firing rate will be affected as in the case of “80/80” paradigm when the PC -> PC Epsp size is 1.5 mV. Apart from this single exception, the peak firing rate showed a linear response. (D) The onset of MC firing was quicker when PC -> MC connection strength was doubled and tripled.

Increasing the PC - PC synaptic strength decreased the attractor duration, owing to increase in firing and faster adaptation and depletion of vesicles (Fig. 4a). The response in that “15/80” paradigm was similar to the network without MCs since 15% convergence of PCs on MCs did not lead to much MC discharge. However, “40/80” and “80/80” paradigms had distinguishable effect due to higher convergence (Fig. 4a). Increasing the PC - MC connection strength showed a similar effect (Fig. 4c), for obvious reasons. When PCs enter the attractor state, their firing rate will be on the increase for about 100 ms, until nonlinearities like synaptic depression and spike-frequency adaptation kicks in bringing the firing rate down. If the discharge of MCs supersedes the above nonlinearities, the peak firing rate will be lowered, otherwise it remains unaffected as shown in Fig. 4c, d. The values that were held constant are shown on the top of each sub-figure.

### Regulation of Attractor Dwell Time

**Figure 5:**
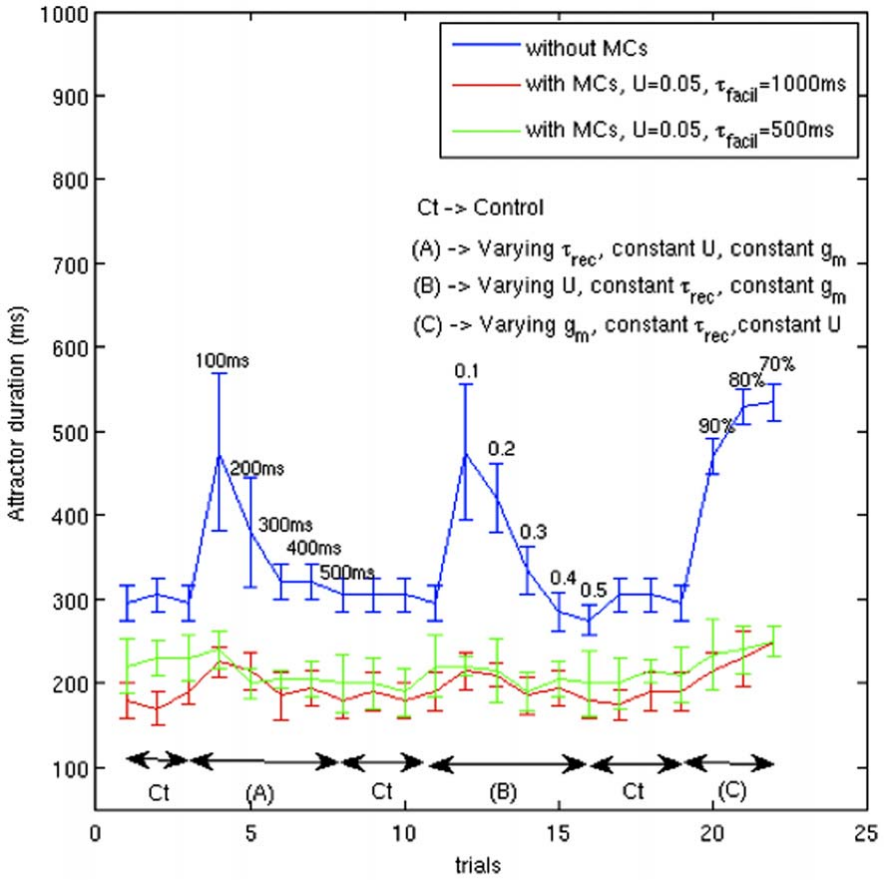
Teasing out the parameters that cause attractor termination. Every point on the figure is an average of the attractor duration of all stored patterns calculated for each trial. The error bar gives their variation in each trial. Interspersed between the three conditions ((A), (B), (C)) are the control conditions (Ct) to show the sensitivity of attractor duration in the absence of MCs (blue) and presence of MCs with different facilitation time constants (red and green). Throughout this analysis, we have used the “40/80” paradigm for the connections between PCs and MCs. The short-term plasticity values at various synapses during ‘Ct’ are given in Table 3. The numbers just above the error bar are the values assumed by the varying quantity during (A), (B) and (C); the values at (C) are the percentage difference from ‘Ct’. In the absence of MCs, the attractor duration is sensitive to the lower values of depression between PC-PC synapses, brought about by lowering ‘U’ (A) or lowering τ_rec_ (B), and changes in *g_M_* of PCs (C), inasmuch as these factors results in increase in PCs firing. The height of the errorbar at the lower values of depression ((A), (B)) is also high implying a large variation in the attractor duration of all stored patterns in every trial. When MCs inhibition is included (red), sensitivity to change in STD and adaptation are minimal, there are no strong peaks apart from trial-to-trial variation of attractor duration. Besides, the presence of MCs inhibition also prevents the scatter of data from its mean value in every trial (note the steady values of green and red errorbars). Decreasing the τ_facil_ of PC-MC synapses (green) shows a similar response.

The attractor state termination could be due to intrinsic neuron properties like adaptation and synaptic depression or due to the presence of MCs inhibition in the network. At this juncture, we were interested to investigate which factors dominate in controlling the attractor state dwell time. To address the effect of synaptic depression, we varied the strength of depression between PC - PC synapses by lowering τ_rec_ that would speed up the rate of recovery of vesicles and by lowering *U* that would decrease the vesicle release probability resulting in lower depression [36]. We controlled the effect of spike-frequency adaptation by down-regulating the conductance of the M-current (*g_M_*). Figure 5 shows the attractor duration calculated for various conditions in the presence and absence of MC inhibition. In the absence of MC inhibition (blue curve), at lower values of τ_rec_ and *U*, PCs discharge more due to lesser depression, leading to an increase in attractor duration in comparison with the control condition. At higher values of τ_rec_ and *U*, the attractor duration attains constancy. In the same vein, lowering *g_M_* of PCs prolongs the attractor state conforming to previous studies [12], [41].

In the presence of MC inhibition (Fig. 5, red curve), the attractor duration is reduced as shown in the control condition ‘C’. However, the lower values of τ_rec_ and *U* causing lesser depression did not prolong the attractor state as it did when the MC inhibition was turned off. Furthermore, the variation in the dwell time at low τ_rec_ and *U*, shown by the height of the error bar, in the presence of MCs is low in comparison to when MCs inhibition was absent. As expected, lowering *g_M_* of PCs in the presence of MCs did not change the attractor duration significantly. Comparing the red curve and green curve in Figure 5 demonstrates that the network shows similar response for a change in τ_facil_. But large changes in PC-MC or MC-PC connection density affected the attractor duration (not shown here). Thus, in this network model the MC inhibition is the dominating factor in regulating the attractor dwell time and it brings more stability at lower values of depression and spike-frequency adaptation. The latter aspect becomes important in the light of heterogeneity in short-term synaptic parameters.

**Figure 6:**
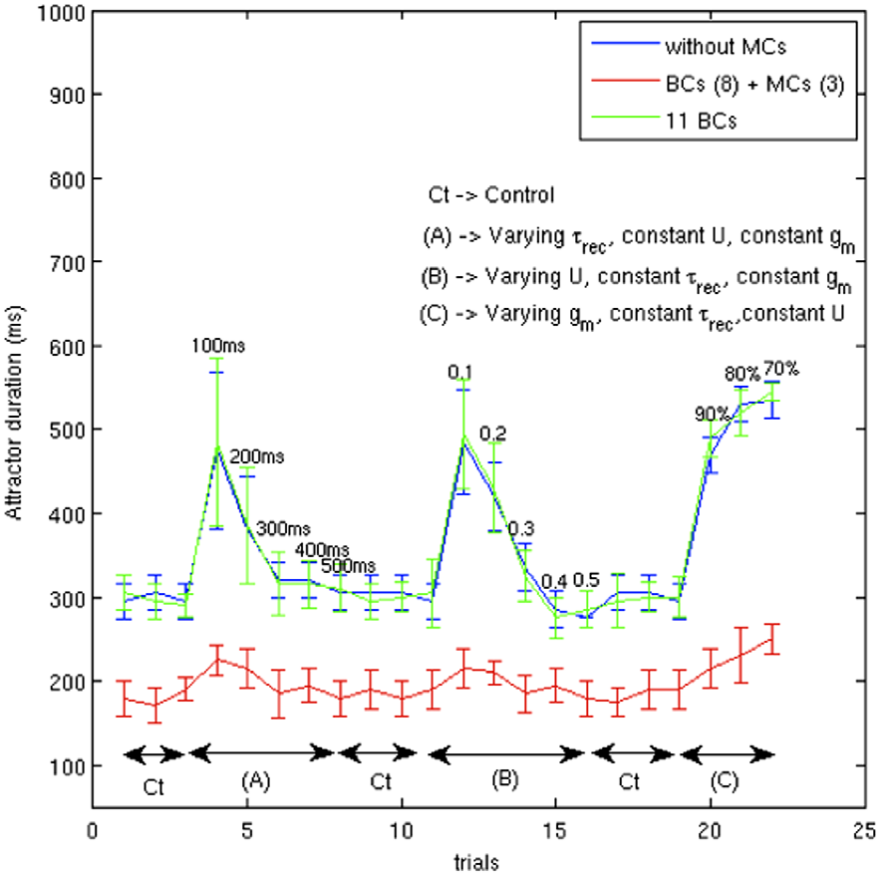
Each hypercolumn contains 8 BC and 3 MC. Here we investigated the sensitivity of the network during different conditions, similar to the ones in Fig. 5, when each hypercolumn has a) 8 BC b) 8 BC + 3 MC and c) 8 BC + 3 BC in total 11 BC. Again, we have used the “40/80” paradigm for the connections between PCs and MCs like in Fig. 5. The response of the network to 8 BC (blue) and 8BC + 3 MC (red) are taken from Fig. 5 (see the blue and red curve there). Conflating the BCs and MCs population, 11 BC, in all hypercolumns doesn’t make the network insensitive to changes in synaptic and cell parameters (green). We observe strong peaks during the lower values of short-term depression and adaptation.

Initially, the network had 8 BCs in every hypercolumn. Then we introduced 3 inhibitory neurons with different synaptic dynamics, MCs, in every hypercolumn. Is the attractor dwell time rigidity to various parameter changes, as shown in Fig. 5, really due to the presence of MCs? In order to address this, we compared the sensitivity of the attractor dwell time when 1) each hypercolumn had 8 BCs “BC (8)” 2) each hypercolumn had 8 BCs and 3 MCs “BC (8) + MC (3)” 3) each hypercolumn had 11 BCs “BC (11)” (Fig. 6). Having BCs alone in the hypercolumn (blue and green) makes the dwell time amenable to changes in short-term synaptic and cell intrinsic parameters. It is only due to the presence of MCs (red) that the dwell time is insensitive to these changes.

## Discussion

In this work, we commenced by modeling the PC-MC microcircuit, as previously shown by Silberberg et al. [22], and reproduced (a) frequency dependent disynaptic inhibition of PCs (b) frequency dependent recruitment of MCs. The model microcircuit contained 3 MCs mediating disynaptic inhibition between 2 PCs. The PC – MC synapses were facilitating and MC – PC synapses were depressing. We stimulated the presynaptic PC with trains of AP at different frequencies and demonstrated increase in amplitude and decrease in latency of disynaptic response of the post-synaptic PC (Fig. 1). Real neurons in the brain, excitatory and inhibitory alike, are constantly bombarded with synaptic inputs causing the membrane potential to fluctuate. In the absence of any jitter, the number of MCs responding to firing of presynaptic PC at different frequencies would remain constant. When a Poisson source was added to all MCs, we observed trial-to-trial variability in the response of MCs. Only one MC fired in most of the trials when the input frequency was 40 Hz, about 2 MCs fired in 90 percent of trials for 50 Hz and all MCs discharged reliably when the input frequency was 70 Hz (Fig. 1). Our model MCs displayed frequency dependent recruitment much similar to slice data and we managed to fit disynaptic response quantitatively using 3 MCs [22], [29].

We then integrated this PC-MC microcircuit in our cortical attractor network model (Fig. 2) [11]–[13]. Amidst the array of phenomena that our network model can exhibit, we focused on the effect of MCs when the network is spontaneously hopping between the attractor (memory) states in the absence of any external input. Such attractor states have been observed in neocortical slices with their active state duration ranging typically from 50 ms to 5 s [16]. In our model, as described below, this dwell time can be controlled by various parameters. Raster plots and average firing rates were used to demonstrate the temporal variation in the firing of BCs and MCs. We showed that BCs that receive depressing synapses has a high firing rate at the beginning of the attractor state which then tapers off. On the other hand, MCs that receive facilitating synapses display a late onset of activation and promote termination of the attractor states owing to their high divergence onto the PCs (Fig. 3). In our model the excitation-inhibition dynamic balance during the attractor state is maintained by the PC – BC pathway until the discharge of MCs tips this balance by elevating inhibition.

Our earlier computational study [12], in agreement with others [9], [53], had demonstrated spike-frequency adaptation and synaptic depression contributing to termination of attractor states. A computational model in the context of relaxation oscillations has recently addressed whether cellular adaptation or synaptic depression affects episode inititation/termination the most [27]. Our attractor dwell time also relies upon those factors in the absence of MCs, but the presence of MCs makes the dwell time impervious to those changes (Fig. 5). Our network model overtly demonstrates that the late onset of MC discharge due to facilitating synapses has a stronger contribution in setting the attractor dwell time and rate of switching between the attractor states compared to spike-frequency adaptation and synaptic depression between PCs.

Introducing three synaptically different interneurons (MC) in our hypercolumn increased the robustness of our network as described above. In order to assay if it is the presence of MCs that lead to increase in robustness, we replaced the three MCs with three BCs in each hypercolumn and performed the sensitivity analysis varying the same set of parameters (Fig. 6). Clearly, the “w/o MC” and “BC(11)” exhibited the same behaviour for changes in synaptic and adaptation parameters. Thus the presence of temporally different interneurons, early-onset BC and late-onset MC, did make the network more robust.

We find gamma oscillations when the network is in the attractor state. We haven’t shown the synthetic LFP spectrograms here but this result could be extended from the analysis made in the previous work [13]. These gamma oscillations in every attractor state are caused by the PC- BC reciprocal circuitry in conformity with the well-established idea that entrainment of PCs by BCs causes gamma oscillations [54], [55]. The dwell time of our attractor states with MC inhibition is about 200 ms, which results in a (5 – 6) Hz state transition frequency. We propose that it is the combined action of BC and MC inhibition that causes this theta/gamma oscillation. With BC inhibition alone attractor switching remains but gets slower (Fig. 5, 6). When we replace all the BCs with MCs, the attractor dynamics were largely disrupted (data not shown). There was a period of no inhibition on all the PCs due to the late onset of MC discharge during which all the patterns were active and this also affected the theta/gamma phenomenon.

When quiescent neocortical slices are bathed in artificial cerebrospinal fluid (ACSF), it induces spontaneous fluctuations between two quasi-stable states knows as UP- and DOWN- states [53]. The stability and repeatability in the patterns of firing during UP states have suggested that they could correspond to attractor states of the network [16]. By using new optogenetic techniques [56]–[58] it should be possible to selectively depolarize or hyperpolarize the different types of inhibitory cells that participate in this activity and investigate the roles of each type. Our model proposes that light-activation of MCs would terminate UP-states while hyperpolarization would extend them. Selective light-induced depression of BCs would likely lead to more vigorous and less distinct oscillatory network activity.

As mentioned in the introduction, a model proposed by Melamed et al. [18] attributes the interaction of excitatory neurons with recurrent excitation and interneurons receiving facilitating synapses as the basis for the generation of slow oscillations in the neocortex. They showed the dependence of network oscillations on the strength of PC-MC synapses corroborating with our study (Fig. 4). However, our study showed no change in the attractor rate switching for any change in the facilitation time constant (Fig. 5), which is not in agreement with their study. The main differences between the two models are the type of neuron model and the division of labour between interneurons. Our spiking model based on the Hodgkin-Huxley formalism utilized two interneurons with temporally disparate workings, whereas their rate-based model only represented the MC interneuron population. Despite these differences, both models pinpoint the decrease in excitation and elevation of inhibition as the most plausible causes of attractor termination.

Some recent experimental studies had addressed the receptive field properties of excitatory and inhibitory cells in layer 2/3 and 4 of mouse visual cortex [59], [60]. The orientation tuning and direction selectivity of pyramidal cells were highly tuned as opposed to basket cells that were largely unselective to any particular orientation concurring well with our modeling study. Interestingly, MCs were also direction selective yet not as highly tuned as PCs and provided delayed inhibition. In the small-scale version of our network presented here, the extent of BC and MC inhibition is the same since each hypercolumn has only 5 minicolumns. But in our full-scale model the number of minicolumns receiving BC inhibition will be more than those receiving MC inhibition since BC inhibition is more laterally spread than MC inhibition in the real cortex. Thus in our full-scale conceptual model, the orientation tuning of MCs would be intermediate between PCs that are highly selective and BCs that are largely unselective, concurring well with the experimental study.

The short-term plasticity parameters used in this model are taken from silent slice data [1], [22], but the cortex is permanently active during awake and sleep states. Thus the presence of ongoing cortical activity might have an impact on short-term plasticity. Some experiments show this to be the case. Synaptic potentials showed a stronger depression in silent slices than in the active cortical network in vivo and in vitro [61], [62]. From our sensitivity analysis, it is clear how attractor dwell times change in relation to short-term depression parameters. Future *in vivo* experiments describing synaptic pathways of excitatory and inhibitory neurons need to take this difference into consideration.
